# Antitumor Effects of Oncolytic Adenovirus-Carrying siRNA Targeting Potential Oncogene EphA3

**DOI:** 10.1371/journal.pone.0126726

**Published:** 2015-05-15

**Authors:** Yali Zhao, Hailiang Li, Ruiqin Wu, Shanhu Li, Peng Wang, Hongtao Wang, Jian Wang, Jianguang Zhou

**Affiliations:** 1 Department of Medical Molecular Biology, Beijing Institute of Biotechnology, Beijing, P. R. China; 2 Astronaut Research and Training Center of China, Beijing, P. R. China; 3 Southern Medical University, Guangzhou, P. R. China; University Hospital of Navarra, SPAIN

## Abstract

Conditionally replicating adenoviruses (CRAds) armed with antitumor transgenes hold promise for cancer treatment. In previous studies, we showed that the 1504-siRNA targeting potential oncogene EphA3 was an efficient therapeutic transgene and that the telomerase reverse transcriptase promoter (TERTp) driving the CRAd was a more advanced generation of CRAd. Therefore, we combined Ad-TERTp-E1A-1504 by inserting 1504-siRNA into the CRAd to study its antitumor effects and mechanism of action, using Ad-TERTp-E1A-NC and nonreplicating adenovirus carrying 1504-siRNA as controls. Cell viability assays and ED_50_ studies of growth inhibition confirmed that Ad-TERTp-E1A-1504 has 3.5- and 1,400-fold greater ability to kill EphA3- and TERT-expressing tumor cells compared to Ad-TERTp-E1A-NC and Ad-ΔE1A-1504, respectively. Also, Ad-TERTp-E1A-1504 had little effect on cells that modestly expressed EphA3 and TERT such as 2BS. The antitumor efficacy of Ad-TERTp-E1A-1504 was also validated *in vivo*. Furthermore, the virus yield of Ad-TERTp-E1A-1504 in C4-2B was ~1,000 times greater than that in 2BS. No obvious differences were observed between Ad-TERTp-E1A-1504 and Ad-TERTp-E1A-NC. Both acridine orange staining and Beclin1 protein measurements indicated that autophagy with Ad-TERTp-E1A-1504 at 5 and 10 MOI was higher than that of Ad-TERTp-E1A-NC. Finally, the classical negatively regulated autophagy signaling pathway, PI3K/AKT/mTOR, was suppressed (reduced phosphorylated form) in contrast to NC, and that this was mediated by 1504-siRNA. Thus, Ad- TERTp-E1A-1504 does not harm normal cells but has dual inhibiting and killing effects on TERT- and EphA3-positive tumor cells, and this effect is mediated by the AKT/mTOR signaling pathway via induction of autophagy. These data may offer a foundation for novel antitumor therapies targeting this mechanism.

## Introduction

EphA3 is recognized as a potential oncogene due to its greater expression or greater incidence of mutation in diverse human tumors, such as hematopoietic and lymphoid tumors, primary leukemia, and T-cell lymphomas, renal and colon carcinomas, malignant melanomas. Furthermore, EphA3 is poorly expressed at normal tissues [1–4], which we observed as well (S1 Fig). EphA3 has important roles in growth and migration/invasion of some cancer cells *in vitro* as well as a role in tumor growth, invasiveness, angiogenesis, and metastasis *in vivo*. In many studies, soluble EphA3 (Fc fusion proteins) and anti-Eph Mabs, agonists and drugs that stimulate Eph receptor degradation were utilized as anti-tumor drugs [5,6]. Recently, small interfering RNAs (siRNA), duplexes to attain sequence-specific RNA knockdown, represent a powerful tool for treating cancer. Previously, a positive correlation between EphA3 expression and Gleason grades in prostate cancer was reported and that EphA3 promotes proliferation and migration of prostate cancer cells was also confirmed [7]. Also, we constructed a plasmid carrying siRNA targeting EphA3, and after 1504-siRNA knockdown of EphA3 expression, we observed growth inhibition of tumors that expressed EphA3 such as C4-2B, HGC27, and HCT116 [8,9]. Thus, 1504-siRNA may be a new therapeutic gene to target for cancer therapy.

In traditional gene therapy, nonreplicative adenoviruses are used as the delivery system. However, short durations and low levels of gene expression remain major challenges for eradicating cancers. In recent years, conditionally replicating adenoviruses (CRAds) that replicate specifically in tumor cells with subsequent oncolysis and release of viral progeny to further infect and destroy neighboring cancer cells have been developed as a gene therapy delivery platform. This technique increases the specificity of the transgene and increases its cancer killing potency [10]. CRAds have undergone two generations of deletion/mutation CRAds and tumor/tissue-specific promotor driving CRAds, respectively. Reverse transcriptase telomerase promoter (TERTp) driving the CRAd is among the most advanced CRAds because it targets approximately 90% of human cancers and has greater potency for replicating in cancer cells and destroying them [11]. Therefore, we inserted 1504-siRNA into TERTp driving CRAd to construct a novel CRAd: Ad-TERTp-E1A-1504, with the intent to more efficiently destroy tumors with less damage to adjacent normal cells. In addition, we also explored the mechanism behind the recombinant CRAd form the perspective of autophagy to understand how future combinations with other therapeutic antitumor drugs might be achieved.

## Materials and Methods

### Ethics Statement

All procedures for animal experiment were approved by the Animal Experiment Committee of the Institute of Biotechnology and performed in accordance with the institution guidelines.

### Cells and cell culture

The human cell lines C4-2B, LNCap (prostate cancer), MGC803, HGC27(gastric carcinoma), SMMC-7721, Bel-7402(hepatic carcinoma), WCY, MCF-7(Breast cancer), H1299, 95D(pulmonary cancer), Hela(cervical cancer), HT29, HCT116(colon carcinoma), LO2(hepatocyte), 2BS(normal lung fibroblast), HEK293(embryonic kidney) and B16(melanoma) origing from mice, were obtained from Nanjing keygen Biotech company. All cell lines were cultured according to the vendor’s instructions.

### Generation of recombinant CRAd and identification

#### Generation of recombinant plasmids (S2 Fig)

pshuttle, pTE-TPE (the E1 region-bearing plasmid, the region containing the promoter of E1A was replaced by TERTp) were kindly provided by Professor Lu from Chinese Center for Disease Control and Prevention [12]. The 1504-siRNA and NC sequence, amplified by PCR from pGP-U6-1504-shRNA and pGP-U6-NC respectively, was first subcloned into pshuttle to form psh-1504-siRNA and psh-NC. Then the sequence of TERTp and E1A and E1B excised from pTE-TPE was subcloned into psh-1504-siRNA or psh-NC to generate psh-TETPE-1504 or psh-TETPE-NC which was recombinated homologously with pAd-easy in *Escherichia coli* BJ5183. The nonreplicative adenovirus, Ad-△E1A-1504 and Ad-△E1A-NC was also made through pAd-easy, pGP-U6-1504-siRNA, and pGP-U6-NC. Each recombinant plasmid was identified by enzyme excision and sequencing accordingly.

#### Generation of recombinant adenoviruses

CRAds and nonreplicating adenoviruses were packaged and amplified in 293 cells according to the manual for the AdEasy system (Cat.240009, Stratagene, La Jolla, CA), and then purified by double CsCl gradient ultracentrifugation. The function of recombinant adenoviruses were identified by cytopathetic effects (CPE), Western blot, or an MTT assay.

### MTT assay

Cells (1×10^4^ cells/well in 96 well plate) infected with adenoviruses or mock infected were treated with MTT (3-[4,5-dimethlythiazol-2-yl]-2, 5-diphenyltetrazolium bromide) as a measurement of the cytotoxicity of adenovirus vectors, absorbance at 495nm was recorded and survival was calculated as a percentage of the measurements taken in untreated cells.

ED50, effective dose for inhibiting cancer cell growth by 50% were calculated through point slope method.

Treatment index (TI) was the ratio of ED50 of adenoviruse for 2BS to that for tumor cells.

### Cytotoxicity assay

Cells were grown subconfluently in 24 well plate and infected with adenoviruses with indicated titers or mock treated for 2 h followed by replacement of infection media with growth media. 3 days post-infection, the cells were stained with crystal violet and quantified.

### Human tumor xenograft model in nude mice

HGC27 cells (5×10^6^cells in 100μl of PBS) were inoculated s.c into the two sides of flanks of the same female BALB/c nude mouse at the age of 4–5 weeks. Treatment with viral constructs was initiated when tumor xenografts reached a diameter of >0.5cm. Six mice were chosen. Each mouse of three received three daily intratumoral injections of Ad-TERTp-E1A-1504 or Ad-TERTp-E1A-NC at 1×10^7^ plaque-forming units (pfu) at one side. Control mouse of the other three received injection of PBS only. Xenograft sizes (width and length) were measured by a vernier caliper twice a week. Tumor volume (V) was calculated by the formula: V = W^2^ x L/2. Animals were euthanized about 30 days after injection. Growth rate = (V-V_0_)/V_0_, (V or V_0_, was volume of xenograft at the end of experiment or beginning volume when it was treated with viruses). The tumor sample was used for protein extraction and western-blot.

### Virus progeny production assay

C4-2B and 2BS cells were seeded in 24-well plates at a density of 1×10^5^ per well and infected with the CRAds at a multiplicity of infection (MOI) of 10. After incubation for 2 h, viruses were removed, and then rinsed twice with culture media containing 2% FBS, and 600μl of fresh media were added to each well. Cells were collected and combined with culture supernatant 24, 48, 72h post-infection. Lysates were prepared by three cycles of freeze-and-thaw and titrated by limiting dilution assay on 293 cells [13].

### Western Blotting

Cells were infected with CRAds or replicative adenoviruses at respective MOIs and were harvested 36~48h post infection, followed by protein expression levels testing. Primary antibodies of E1A and EphA3 were obtained from Santa Cruz Biotechnology, and that of AKT, pAKT, mTOR, pmTOR, 4EBP1, p4EBP1, Beclin1, and p62 were from cell signaling corporation.

### Autophagy assay

For fluorescent microscope analyzing, C4-2B cells were grown in square coverslips 24 h before infection. Next day, cells were infected with 1, 5, 10 MOI of Ad-TERTp-E1A-1504 or Ad-TERTp-E1A-NC or were mock infected. After 2 h absorption, unbound viruses were removed and fresh portion of growth media were added. At 40 h post-infection, the cells were rinsed with PBS and stained with 1 μg/mL acridine orange (Sigma-Aldrich) for 20 min at 37°C following a rinse with PBS. Thereafter 10 visual fields of cells selected randomly were taken pictures under a fluorescent microscope and Photometrics Cool Snap HQ camera connected to a Delta vision RT Restoration Imaging System (Applied Precision), and were analyzed through photoshop, counting the red-colored cells and total cells respectively, and calculating the percentage of the red-colored cells. For flow cytometer analyzing, the cells in six well plates were infected with CRAds, then detached with trypsin and stained with Acridine as above. Finally cells were analyzed using the PerCP-Cy5.5-A channel with a BD FACS Canto II (BD Biosciences) flow cytometer.

### Statistics

Data are presented as mean values ± standard deviation. Statistical difference was assessed with a two-tailed Student’s t-test. A p-value of <0.05 was considered significant.

## Results

### Construction and identification of Ad-TERTp-E1A-1504

**Fig 1:**
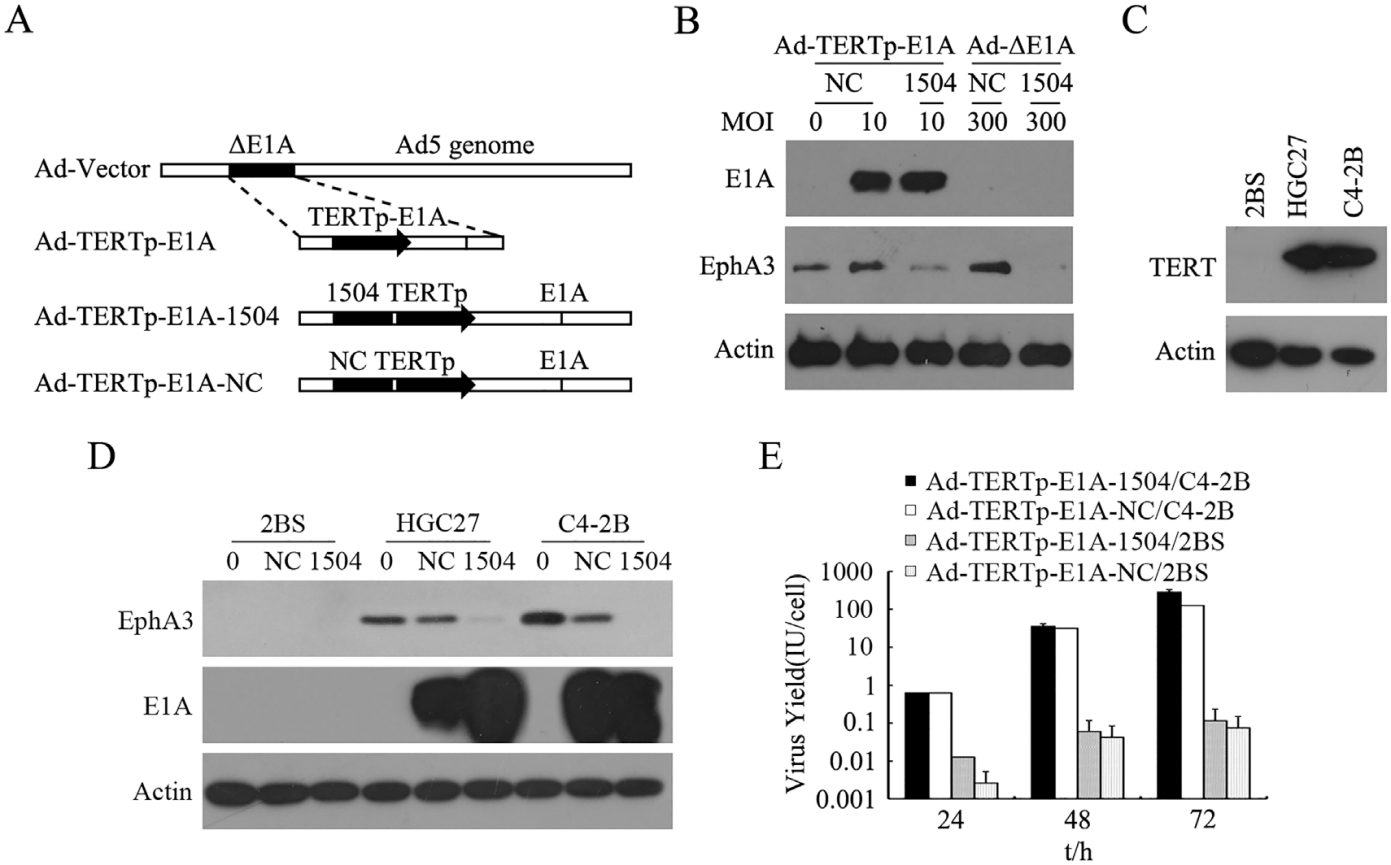
Traits of Ad-TERTp-E1A-1504 and Ad-TERTp-E1A-NC. A: illustration of E1A-completent adenoviruses. 1504-siRNA was incorporated into TERTp driving the E1A adenovirus vector, and Ad-TERTp-E1A-NC was produced by replacing 1504-siRNA with a negative control sequence. B: EphA3 and E1A expression after viral infection of C4-2B. C: TERT expression in 3 cells lines (Western blot) D: EphA3 and E1A expression viral infection of C4-2B, HGC27, and 2BS. E: Replication of CRAds in TERT-positive tumor cell lines and TERT-negative cells. Viral yield in C4-2B increased significantly with greater exposure and viral yield in 2BS cells increased slightly 24 to 72 h after infection.

A diagram of E1A-competent adenoviruses showed that 1504-siRNA was incorporated into TERTp driving the E1A adenovirus vector, while Ad-TERTp-E1A-NC was produced by replacing 1504-siRNA with a negative control sequence (Fig 1A). The structure was confirmed by experiments as follows:

Protein expression data (Fig 1B and 1C) show that only TERT-mediated CRAds elevated E1A expression in cells that greatly expressed TERT (Fig 1D), such as C4-2B and HGC27. Replication-defective adenoviruses (MOI of 300) did not increase E1A expression in C4-2B cells and TERT-mediated CRAds did not induce E1A expression increases in TERT-negative cells.

Fig 1E shows that the viral yield in C4-2B increased significantly (from 0.6 to 100 IU/cell) whereas viral yield in 2BS cells increased slightly (from 0.01 to 0.1 IU/cell). Thus, CRAds could replicate specifically in tumor cells. Also, the replication capability of Ad-TERTp-E1A-1504 was similar to that of Ad-TERTp-E1A-NC, indicating that the enhanced antitumor efficacy of Ad-TERTp-E1A-1504 compared to Ad-TERTp-E1A-NC was not due to replication-inducing tumor cell lysis.

### Effects of Ad-TERTp-E1A-1504 on telomerase-positive and -negative cells

**Fig 2:**
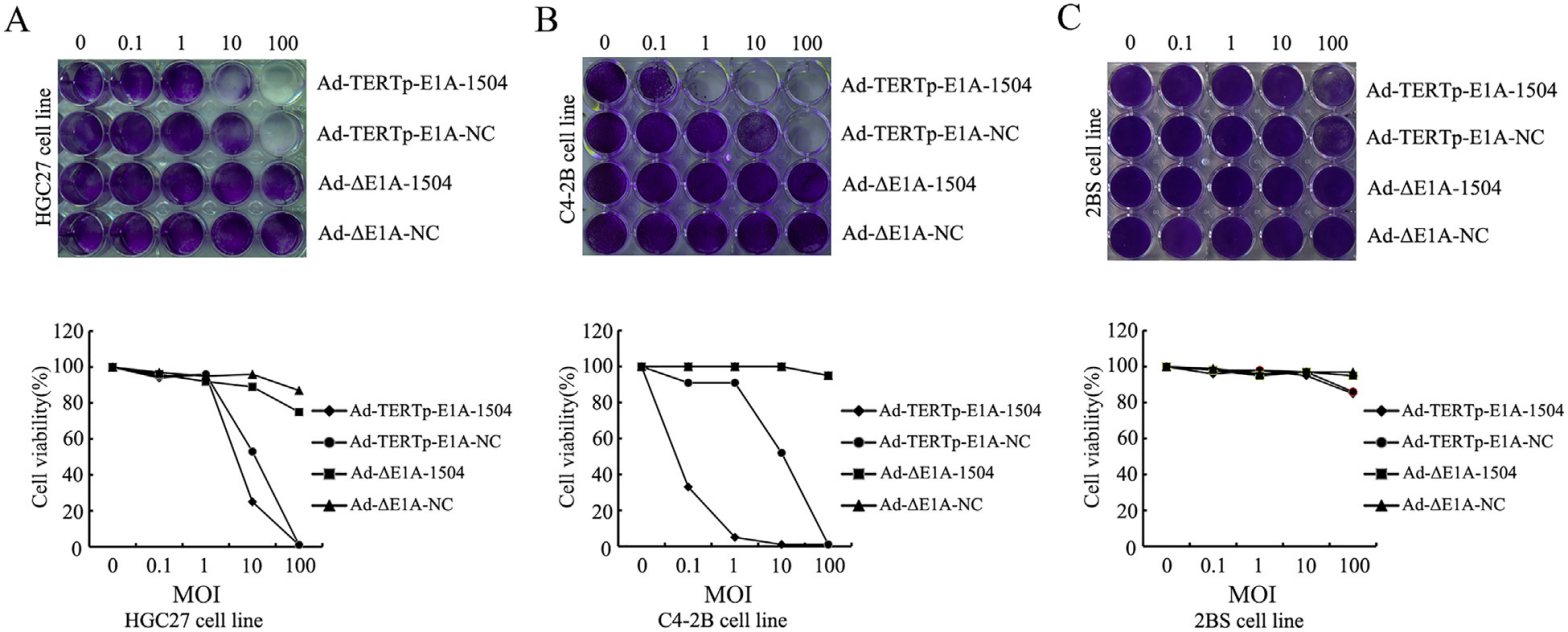
Ad-TERTp-E1A-1504 enhances oncolytic potency. C4-2B, HGC27, and 2BS were infected with Ad-TERTp-E1A-1504, Ad-TERTp-E1A-NC, Ad-△E1A-1504, or Ad-△E1A-NC at the indicated MOIs. After 3 days, adherent cells were stained with crystal violet and quantified. Data represent examples of two independent sets of experiments. A-B: Ad-TERTp-E1A-1504 killed almost all tumor cells at 1–10 MOI, however Ad-TERTp-E1A-NC had the same effect at 100 MOI and Ad-ΔE1A-1504 or Ad-ΔE1A-NC killed fewer cells at 100 MOI. C: Compared with tumor cell lines, 2BS was less sensitive to Ad-TERTp-E1A-1504 (few cells died at 100 MOI).

Two human tumor cell lines highly expressing EphA3 and TERT(C4-2B and HGC27 cell) and cell line that expressed little EphA3 and TERT (2BS cell) were infected with Ad-TERTp-E1A-1504, Ad-TERTp-E1A-NC, or Ad-ΔE1A-1504 or Ad-ΔE1A-NC at the indicated titers. Then, 3 days after infection, Ad-TERTp-E1A-1504 killed most of the tumor cells (1–10 MOI) but Ad-TERTp-E1A-NC had the same effect at 100 MOI. Ad-ΔE1A-1504 or Ad-ΔE1A-NC killed fewer cells at 100 MOI (Fig 2A and 2B). Compared with tumor cell lines, 2BS was less sensitive to Ad-TERTp-E1A-1504; few cells died at 100 MOI (Fig 2C). Thus, Ad-TERTp-E1A-1504 offered dual killing effects for cell lines highly expressing TERT and EphA3 and had little effect on negative cell lines.

### Inhibition effects of Ad-TERTp-E1A-1504 on C4-2B cells

**Fig 3:**
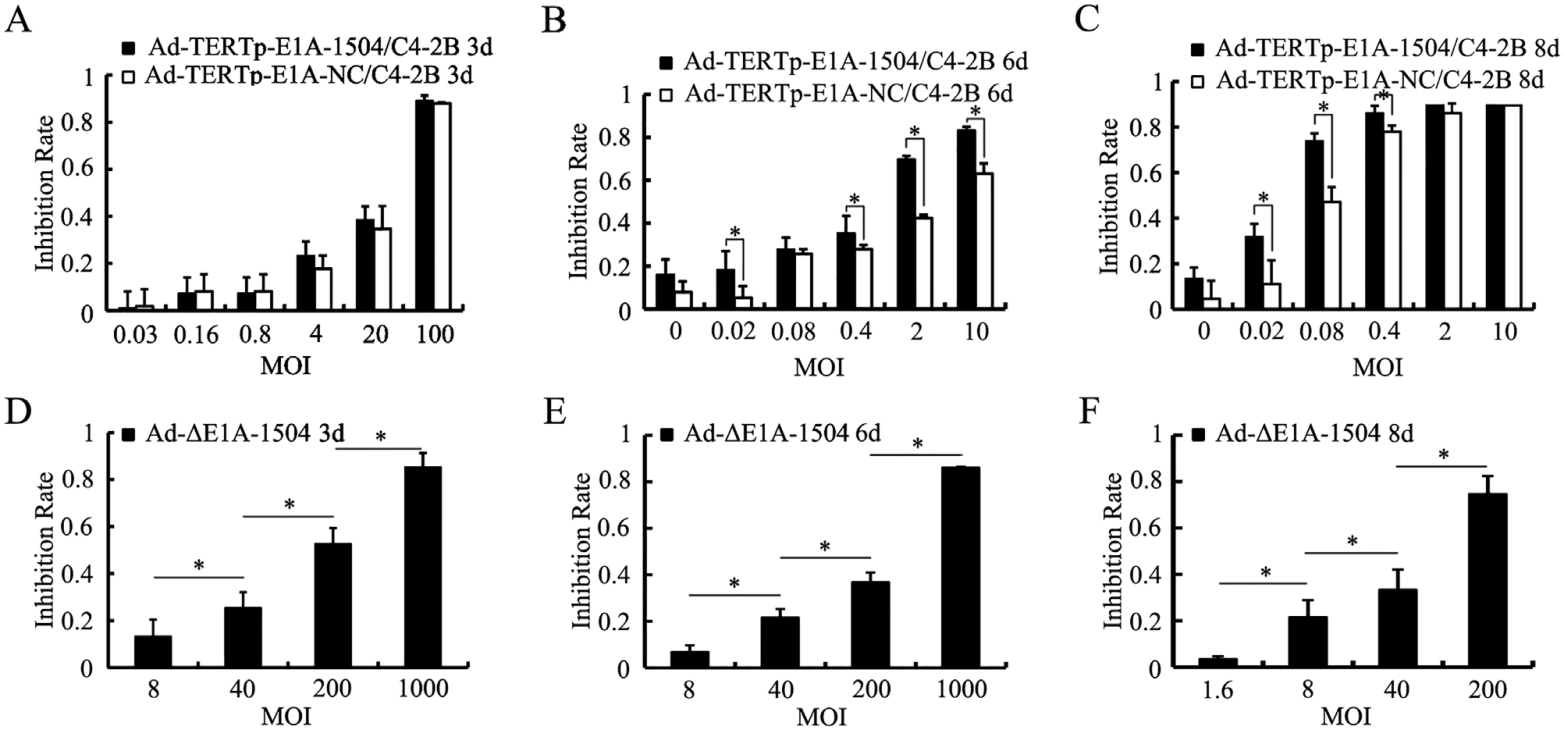
Inhibition effects of Ad-TERTp-E1A-1504 on C4-2B cells. Inhibition of proliferation of C4-2B cells was measured with MTT. A, B and C: inhibition resulting from Ad-TERTp-E1A-1504, Ad-TERTp-E1A-NC at different MOIs 3, 6, and 8 days after infection. They showed that the longer infection duration, the lower MOIs were needed to attain high inhibition rate, and MOIs of Ad-TERTp-E1A-1504 was lower than that of Ad-TERTp-E1A-NC. For example, to attain inhibition rate of 60% MOIs for Ad-TERTp-E1A-1504 and NC were 100 and 100(3 days), 2 and 10(6 days), 0.08 and 0.4(8 days) respectively. D, E, F: inhibition from Ad-ΔE1A-1504 at different MOIs at 3, 6, and 8 days after infection. Increasing infection duration and greater MOI did not inhibit proliferation as impressively as Ad-TERTp-E1A-1504 and Ad-TERTp-E1A-NC. *, P<0.05.

MTT experiments revealed that the longer infection duration, the lower MOIs were needed to attain high inhibition rate, and MOIs of Ad-TERTp-E1A-1504 was lower than that of Ad-TERTp-E1A-NC. For example, to attain inhibition rate of 60% MOIs for Ad-TERTp-E1A-1504 and NC were 2 and 10(6 days), 0.08 and 0.4(8 days) respectively. Ad-TERTp-E1A-1504 was more inhibitory than Ad-TERTp-E1A-NC (Fig 3A–3C), whereas inhibition with Ad-ΔE1A-1504 was less than that of Ad-TERTp-E1A-1504 and Ad-TERTp-E1A-NC (Fig 3D–3F).

### Inhibition effects of Ad-TERTp-E1A-1504 on TERT^+^EphA3^-^ cell line (293T) and TERT^-^EphA3^-^ cell line (2BS)

**Fig 4:**
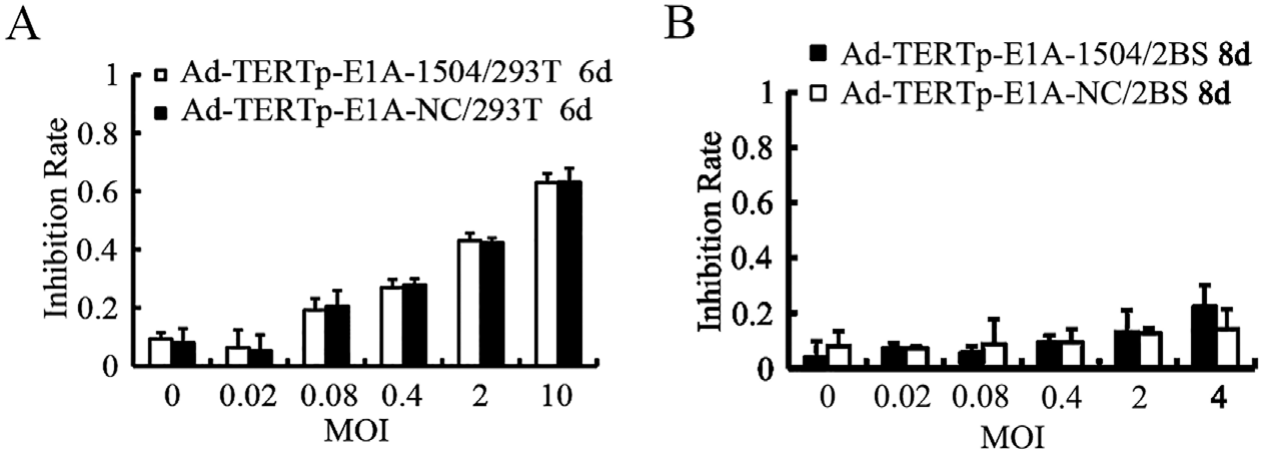
Inhibition effects of Ad-TERTp-E1A-1504 on TERT^+^EphA3^-^ cell lines(293T) and TERT^-^EphA3^-^ cell lines(2BS). Proliferation inhibition was measured with MTT assay. A：With increasing of MOIs of Ad-TERTp-E1A-1504 and Ad-TERTp-E1A-NC, the inhibition efficiency of 293T cells was increased, but no significant difference between both of the CRAds. B: Ad-TERTp-E1A-1504 and Ad-TERTp-E1A-NC did not inhibit 2BS growth at each MOI.

Fig 4A and 4B indicated that with increasing of MOIs of both Ad-TERTp-E1A-1504 and Ad-TERTp-E1A-NC, there was no significant difference of the inhibition on 293T cells. Furthermore, both Ad-TERTp-E1A-1504 and Ad-TERTp-E1A-NC did not inhibit 2BS growth with increasing MOIs.

### ED_50s_ of Viruses and TI of Ad-TERTp-E1A-1504

The ED_50_ quantifies inhibition, and these values for C4-2B and 2BS appear in Table 1. ED_50_ decreased with increasing infection time, and ED_50_ values increased in the order of Ad-TERTp-E1A-1504, Ad-TERTp-E1A-NC, and Ad-ΔE1A-1504. Furthermore, the antitumor efficiency of Ad-TERTp-E1A-1504 at 8 days postinfection was stronger than Ad-ΔE1A-1504 and Ad-TERTp-E1A-NC, by 1,400 and 3.5 times respectively.

**Table 1:** ED50 of adenovirus for inhibiting proliferation and TIs.

Adenoviruses	ED50 of C4-2B		ED50 of 2BS	TI
	3d	8d	8d	
Ad-TERTp-E1A-1504	11.99	0.04	10.96	274.00
Ad-TERTp-E1A-NC	12.94	0.14	11.70	83.60
Ad-ΔE1A-1504	136.00	56.34	371.5	6.60

The ED50 values listed in the table were calculated with point slope method. ED50s were decreased with the increasing of infection duration for the same batch of experiment and ED50 value was decreased by the sequence of Ad-ΔE1A-1504, Ad-TERTp-E1A-NC and Ad-TERTp-E1A-1504. TI was the ratio of ED50 of adenoviruse for 2BS to that for tumor cells. TI of Ad-TERTp-E1A-1504 was higher than that of Ad-TERTp-E1A-NC and Ad-△E1A-1504.

Table 1 depicts TI of C4-2B normalized by the normal cell line. We observed that at 8 days postinfection, TI of Ad-TERTp-E1A-1504 was 274.0 higher than that of Ad-TERTp-E1A-NC(83.6) and Ad-ΔE1A-1504(6.6), suggesting greater safety and clinical utility compared to Ad-TERTp-E1A-NC and Ad-ΔE1A-1504.

### Antitumor activity *in vivo*

**Fig 5:**
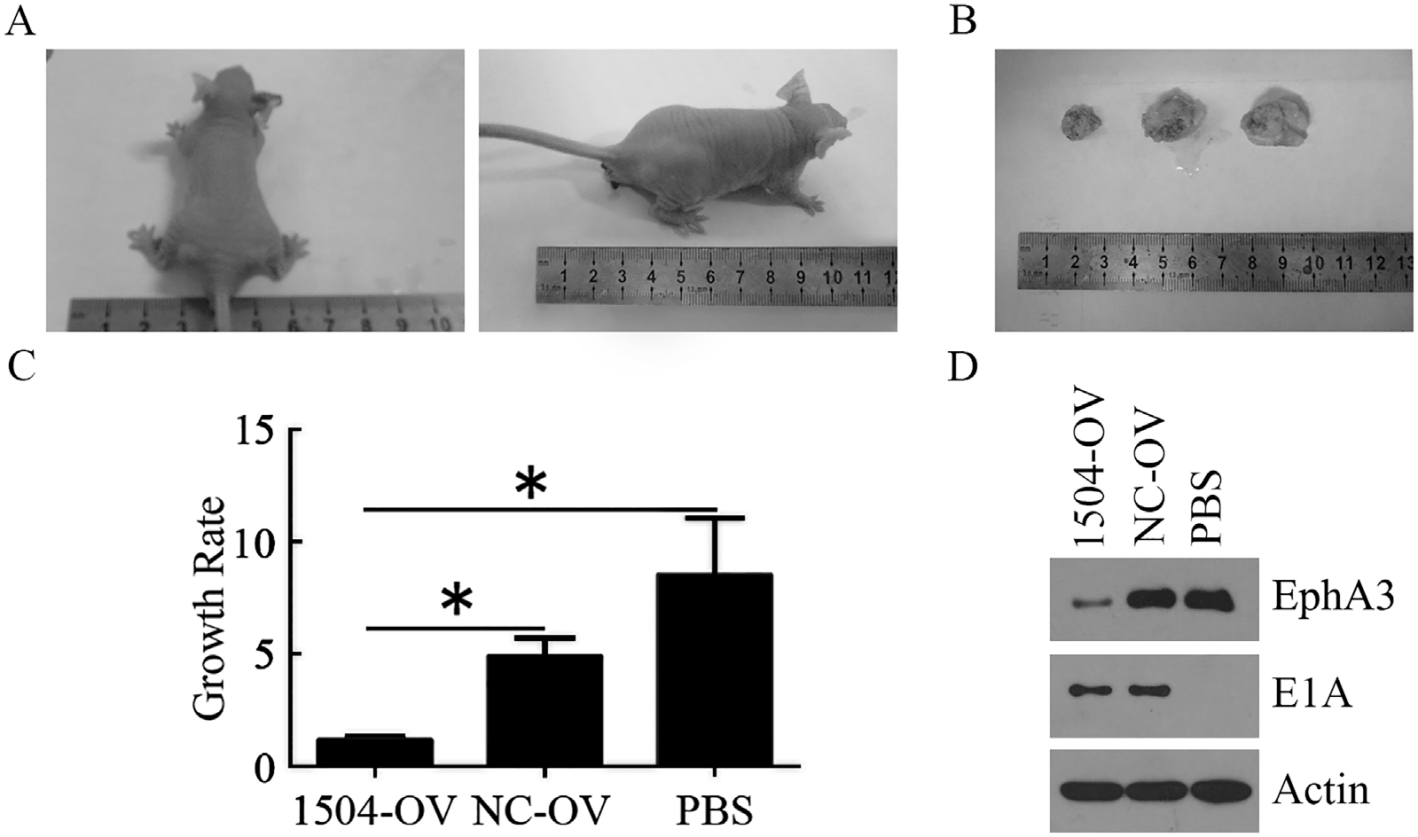
Enhanced tumor growth inhibition of HGC27 xenograft by Ad-TERTp-E1A-1504. HGC27 tumor xenografts were induced in balb/c nude mice by s.c. injection of 5×10^6^cells. Three daily injections of Ad-TERTp-E1A-1504, Ad-TERTp-E1A-NC(both at 1×107pfu) or PBS were given intratumorally when the tumor xenogaft reached a diameter of >0.5cm. A: Representive xenografts when they were first treated with virus or PBS. The volume of them was similar. B-C: Representive xenografts euthanized. The volume of xenograft treated with Ad-TERTp-E1A-1504 was smaller than that with Ad-TERTp-E1A-NC and PBS in order. Growth rate was represented with mean±SE(n = 3). D: Western blot analysis of the expressions of E1A and EphA3 in xenograft tumor tissue treated with Ad-TERTp-E1A-1504, Ad-TERTp-E1A-NC and PBS.

The *in vivo* antitumor efficacy of Ad-TERTp-E1A-1504 was examined with a HGC27 xenograft model in nude mice (Fig 5) and we observed that Ad-TERTp-E1A-1504 at 1×10^7^pfu significantly inhibited growth of xenograft compared with Ad-TERTp-E1A-NC and PBS. Western blot analyses showed that E1A expression was observed in xenografts treated with Ad-TERTp-E1A-1504 and Ad-TERTp-E1A-NC, while EphA3 expression decreased in xenografts treated with Ad-TERTp-E1A-1504 only.

### CRAds induced autophagy in TERT-positive tumor cells

**Fig 6:**
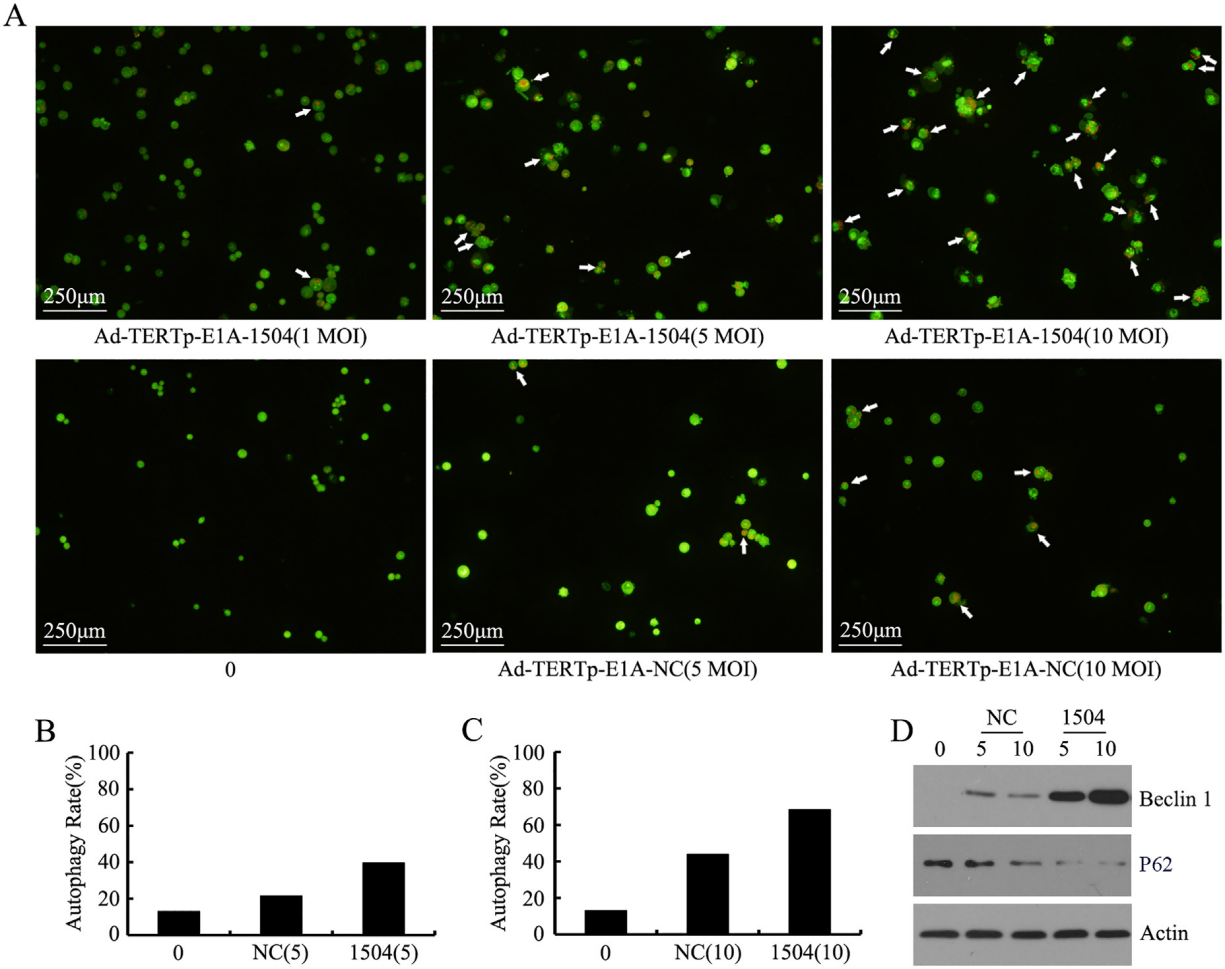
CRAds-induced autophagy in TERT^+^EphA3^+^ tumor cells. At 40 h postinfection (1–10 MOI) with CRAds, C4-2B cell autophagy was measured. A, B, C: autophagosomes were stained with arcridine orange and observed microscopically. Autophagosomes are red vesicles in the cytoplasm. Bar = 40 Am. Autophagy increased with increasing infective dose. Autophagy caused by Ad-TERTp-E1A-1504 (1, 5, and 10 MOI) exceeded that of Ad-TERTp-E1A-NC at the same MOI. Autophagy of CRAds at 5 and 10 MOI exceeded that of control and autophagy at 10 MOI were greater than that at 5 MOI. D: Beclin1 and p62 protein was measured with Western blot after treatment with Ad-TERTp-E1A-1504 and Ad-TERTp-E1A-NC.

Autophagosomes appeared in cells, and autophagy increased with increasing infectivity (Fig 6A–6C). Autophagy caused by Ad-TERTp-E1A-1504 at 1, 5, and 10 MOI were 21.52, 39.60, and 68.35% respectively; and autophagy caused by Ad-TERTp-E1A-NC at 5 and 10 MOI were 21.60 and 43.88%. Autophagy of CRAds at 5 and 10 MOI were greater than control and autophagy at 10 MOI exceeded that at 5 MOI. Data from flow cytometry(Table 2) confirmed these results: Ad-TERTp-E1A-1504 induced more autophagy than Ad-TERTp-E1A-NC.

**Table 2:** The increase of autophage rate determined by flow cytometry after cells were infected with CRAds.

CRAds	Increase rate of autophage(%)	
	5MOI	10MOI
Ad-TERTp-E1A-1504	**56.3**	**37.9**
Ad-TERTp-E1A-NC	**49.9**	**14.8**

An autophagy test was performed by flow cytometry and this was performed by counting autophage-containing cells and dividing this number by counted control cells. Autophagy caused by Ad-TERTp-E1A-1504 exceeded that of Ad-TERTp-E1A-NC at the same MOI.

The autophagy protein marker Beclin1 and p62 was measured by Western blot and the result showed that Beclin1 expressed higher at 5 and 10 MOI of Ad-TERTp-E1A-1504 treatment than that of Ad-TERTp-E1A-NC while p62 showed lower expression. These data indicated that autophagy was the major pathway for CRAds-induced tumor cell killing and was the reason that Ad-TERTp-E1A-1504 could kill more tumor cells than NC (Fig 6D).

### The effect of CRAds on the AKT signal pathway in TERT positive tumor cells

**Fig 7:**
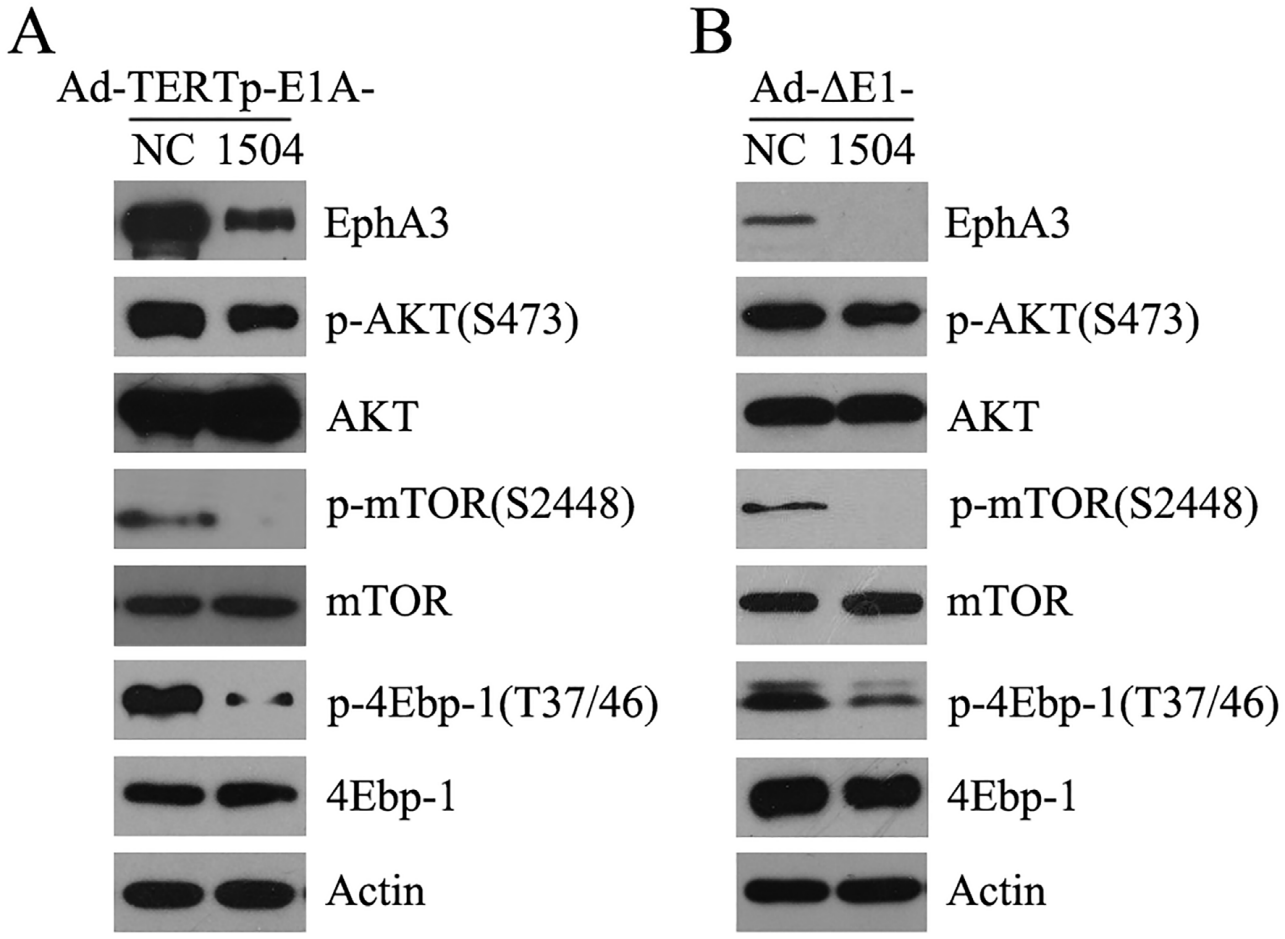
The effect of CRAds on the AKT signal pathway in TERT^+^EphA3^+^ tumor cells. A: C4-2B cells were infected with Ad-TERTp-E1A-1504 and Ad-TERTp-E1A-NC (10 MOI) and 2 days after infection the AKT/mTOR pathway was detected by Western blot. p-mTOR, pAKT, and p4Ebp-1 were downregulated by Ad-TERTp-E1A-1504 compared with NC. B: HGC27 cells were infected with Ad-ΔE1-1504 and Ad-ΔE1-NC (400 MOI) and 2 days after infection the AKT/mTOR pathway was detected by Western blot. p-mTOR, pAKT, and p4Ebp-1 were downregulated by Ad-TERTp-E1A-1504 compared with NC.

Western blot confirmed that Ad-TERTp-E1A-1504 and Ad-ΔE1-1504 significantly suppressed EphA3 and AKT, mTOR and 4-ebp1 phosphorylation compared with Ad-TERTp-E1A-NC and Ad-ΔE1-NC, respectively, in TERT- and EphA3-positive tumor cells. Thus, 1504-siRNA repressed the autophagy feedback signaling pathway to activate autophagy, and this was why Ad-TERTp-E1A-1504 had greater anti-tumor efficacy than Ad-TERTp-E1A-NC Fig 7A and 7B).

## Discussion

Eph receptors and ephrins have been shown to affect the growth and migration/invasion of cancer cells in culture as well as modify tumor growth, invasiveness, angiogenesis, and metastasis *in vivo*. However, Eph signaling activities in cancer appear to be complex, and are characterized by puzzling dichotomies. For example, the EphA3 mutation in lung tissue promoted lung cancer, indicating non-mutated EphA3 inhibited cancer [14]. Still, EphA3 is reported to be an oncogene [4,7]. In our previous work, 1504-siRNA silencing of EphA3 was potent tumor suppressor via the classic tumorgenesis pathway (AKT signaling pathway) [8,9]. However, the method for delivering this gene for treating cancer was uncertain. Because tumor-specific oncolytic adenovirus can amplify many times in infected cancer cells and dissolve them, a TERT promoter driven CRAd was regarded to be the most widely targeting tumor (90% of tumor) and a more potent replicating CRAd [11]. Thus, we used a TERT promoter driven CRAd as a vector to carry 1504-siRNA.

1504-siRNA was constructed into TERTp specifically regulated oncolytic adenoviruses to produce Ad-TERTp-E1A-1504. CRAds were identified by increased E1A expression and decreased EphA3 expression (Fig 1) EphA3- and TERT-positive cell lines (Fig 1B and 1C), in addition to no E1A expression in negative cells. Replication ability of CRAd in TERT-positive cells was confirmed via selectively yielding CRAd in C4-2B cells.

High antitumor efficiency specifically in TERT- and EphA3-positive cells of Ad-TERTp-E1A-1504 was verified by cell killing and MTT experiments. Antitumor efficiency was better than other TERTp driven CRAds. Ad-CD80-TPE-GM, with the granulocyte-macrophage colony-stimulating factor (GM-CSF) gene and CD80 inserted into a TERTp driven CRAd had an oncolytic effect at an MOI of 0.1, and killed ~80% TERT-positive tumor cells within 7 days at an MOI of 1 [15]. Our data showed that Ad-TERTp-E1A-1504 killed ~80% of C4-2B cells (Fig 3B) within 6 days at MOI of 0.1

The antitumor efficiency of Ad-TERTp-E1A-1504 exceeded that of Ad-TERTp-E1A-NC and Ad-ΔE1A-1504. A cell killing assay indicated that at the same MOI Ad-TERTp-E1A-1504 was more oncolytic to C4-2B than Ad-TERTp-E1A-NC and Ad-ΔE1A-1504 suggesting that Ad-TERTp-E1A-1504 had dual killing effects in cells that greatly expressed TERT and EphA3 (Fig 2). MTT data agreed with this. Ad-TERTp-E1A-1504 significantly inhibited growth of C4-2B (Fig 3). The ED_50_ of Ad-TERTp-E1A-1504 for C4-2B was 1/3.5 as much as that of Ad-TERTp-E1A-NC, and was 1/1400 as much as that of Ad-ΔE1A-1504 and this antitumor potential difference increased with prolonged infection (Table 1). Our in vivo experiment also confirmed that Ad-TERTp-E1A-1504 injection resulted in significant reduction of primary tumor size than Ad-TERTp-E1A-NC. The results may be explained by replication and oncolysis because Ad-ΔE1A-1504 had no replicating ability and 1504-siRNA was weaker at inhibiting tumors with prolonged infection time. In contrast, both Ad-TERTp-E1A-1504 and Ad-TERTp-E1A-NC could replicate in tumor cells and offer more oncolysis. Moreover, replication of Ad-TERTp-E1A-1504 resulted in increasing copy number of 1504-siRNA which could inhibit tumor proliferation. Therefore, Ad-TERTp-E1A-1504 was more efficacious at eradicating tumors than Ad-TERTp-E1A-NC.

Ad-TERTp-E1A-1504 selectively killed TERT-positive cells more so than negative cells, as confirmed by a cell killing assay. Ad-TERTp-E1A-1504 killed almost all TERT-positive tumor cells, C4-2B and HGC27 at 0.1–10 MOI at 3 days postinfection, but fewer TERT-negative cells were killed (2BS), even at 100 MOI.

The therapeutic index (TI) can offer data about clinical utility. An antitumor drug with a greater TI may be safer and more appropriate for clinical use. Thus, we measured ED_50s_ of diverse viruses in normal cells (2BS) and tumor cells (C4-2B) to obtain a TI. The TI of Ad-TERTp-E1A-1504 at 8 days postinfection was 3.5, 41 times greater than Ad-TERTp-E1A-NC and Ad-ΔE1A-1504, indicating that Ad-TERTp-E1A-1504 had more clinical utility.

The mechanisms of tumorigenesis and tumor development are perplexing. Single treatments have limited effects against tumors, so combinations of antitumor treatments are needed. This means that we must understand each antitumor mechanism of action to design the most optimal combinations. From our study, Ad-TERTp-E1A-1504 with CRAd armed with 1504-siRNA, was more efficacious for killing tumor cells than Ad-TERTp-E1A-NC, with a simple CRAd without the treatment gene. CRAd can execute oncolysis by varied mechanisms. First, CRAds induce anti-tumoral immunity to kill tumor cells directly or to enhance the sensitivity of infected cells to cytokines such as TNF or IFN which in turn accelerate lysis of the infected cells. Next, CRAds replicate and enter tumor cells to lyse their membranes and cause cell death. Some toxic proteins are produced during replication and these can directly kill cells via necrosis or apoptosis or autophagy [16]. Apoptosis is largely the result of caspase-mediated destruction of the cellular structure [17]. Clearly, CRAds caused cell death through many pathways, including apoptosis, autophagy and necrosis, which was dependent on the cell type and the CRAds, viral dose and infection time. Here, we only focused on measuring cell death.

Because autophagy was an initial method of cell death, we studied the effects of Ad-TERTp-E1A-1504 and Ad-TERTp-E1A-NC on autophagy. Theoretically autophagy is caused by metabolic stress and CRAds provide this stress by generously replicating in tumor cells Research shows that TERTp driven CRAd caused autophagy. Other work suggests that certain drug/OV combinations induce autophagy and that intact autophagy pathways do it [18,19]. In our study, 40 h after Ad-TERTp-E1A-1504(1, 5, and 10 MOI) and Ad-TERTp-E1A-NC(5 and 10 MOI) infection of C4-2B cells, autophagy increased compared to control. Ad-TERTp-E1A-1504 had dose-effect response for autophagy and this was greater than that of Ad-TERTp-E1A-NC at the same dose. Furthermore, Beclin1 in Ad-TERTp-E1A-1504-infected C4-2B cells was higher than that in Ad-TERTp- E1A-NC-infected cells. Beclin 1 and P62, autophage marker proteins, were positively and negatively related to autophage respectively [20–22]. Low expression of Beclin1 in 115 node-positive colon cancer specimens was associated with a significantly worse 5-year overall survival (47% versus 67%), which meant that reducing Beclin1 expression promoted cell proliferation and increasing Beclin1 inhibited tumor development. In our research CRAds-induced Beclin1 increases and p62 decreases in expression agreed with our autophagy data, suggesting that CRAd killing of tumor cells was a major pathway, and that Ad-TERTp-E1A-1504 had more potential for killing tumors than Ad-TERTp-E1A-NC.

Because the typical autophagy negative pathway PI3K/Akt/mTOR should participate in autophagy, we investigated whether Ad-TERTp-E1A-1504 inhibited the pathway more than Ad-TERTp-E1A-NC. After 5 or 10 MOI of Ad-TERTp-E1A-1504 and Ad-TERTp-E1A-NC was used, respectively, to infect C4-2B cells, both EphA3 and phosphorylated AKT, mTOR, 4EBP1 were inhibited via 1504-siRNA, confirming that the AKT signal pathway was inhibited in our previous experiments [9,10].

In summary, Ad-TERTp-E1A-1504 did not harm TERT-negative cells but was oncolytic and inhibited TERT- and EphA3-positive tumor cells, which was also validated in vivo. Antitumor effects of Ad-TERTp-E1A-1504 was 1,400-fold that of Ad-ΔE1A-1504, and 3.5-fold that of Ad-TERTp-E1A-NC, and these effects were mediated by autophagy via inhibition of the AKT/mTOR cell signaling pathway. We created a novel recombinant oncolytic adenovirus and offer an innovative method for personal virus-gene design. However, we lacked comparisons with other antitumor drugs as well as investigations on the safety of this novel therapy.

## Supporting Information

S1 Fig Selection of EphA3-highly expressing cell lines. To select EphA3-highly expressing cell lines, EphA3 protein was measured among fourteen cell lines of seven tumor types, C4-2B, LNCaP of prostate cancer, HGC27, MGC803 of gastric carcinoma, WCY, MCF7 of mammary adenocarcinoma, H1299, 95D of lung cancer, SMMC-7721, Bel-7402 of hepatoma carcinoma, Hela of cervical cancer, HT29, HCT116 of colon carcinoma originating from human, B16 of melanoma originating from mice and one normal cell line, LO2. After three batches of experiments C4-2B, HCT116, Bel-7402, SMMC-7721, MCF7, WCY, HGC27 cell lines were confirmed to have more EphA3 protein (S1 Fig). No lung cancer cell lines highly expressed EphA3, confirming that EphA3 was a tumor suppressor gene in lung cancer [17].
(TIF)

S2 Fig Construction maps of plasmids. (A) Plasmid maps of pshuttle-1504, pU6-1504-siRNA, and pshuttle. 1504-siRNA was amplified by PCR with pU6-1504-siRNA as the template using T7 and T3 primers. PCR production cut with KpnI and pshuttle cut with KpnI and EcoRI were ligated to produce pshuttle-1504 that contains 1504-siRNA (5’GCG GTC AGC ATC ACA ACT AAT 3’). (B) Plasmid maps of pshuttle-1504-TETPE and pTE-TPE. pTE-TPE containing the total E1 region except for the promoter of E1A and the promoter of telomerase reverse transcriptase (TERTp) and pshuttle-1504 were all digested with MfeI and NotI and then recovered portions were ligated to generate pshuttle-1504-TETPE which contains 1504-siRNA and the TERTp driven E1A region. (C) Plasmid Maps of pAdEasy-1 and pAd-1504-TETPE. pshuttle-1504-TETPE linearized with PmeI and pAdEasy were mixed and cotransformed into competent BJ5183 cells to produce pAd-1504-TETPE, which contains 1504-siRNA, TERTp, E1A and an Ad backbone.
(TIF)

## References

[1] Hafner C, Schmitz G, Meyer S, Bataille F, Hau P, Langmann T, et al (2004) Differential Gene Expression of Eph Receptors and Ephrins in Benign Human Tissues and Cancers. Clin Chem 50: 490–499. 14726470 10.1373/clinchem.2003.026849

[2] Wimmer-Kleikamp SH, Lackmann M (2005) Eph-modulated cell morphology, adhesion and motility in carcinogenesis. IUBMB life 57: 421–431. 16012051 10.1080/15216540500138337

[3] Ding L, Getz G, Wheeler DA, Mardis ER, McLellan MD, Cibulskis K, et al (2008) Somatic mutations affect key pathways in lung adenocarcinoma. Nature 455:1065–1075. 10.1038/nature07423 PMC2694412 18948947

[4] Smith LM, Walsh PT, Rüdiger T, Cotter TG, Mc Carthy TV, Marx A, et al (2004) EphA3 is induced by CD28 and IGF-1 and regulates cell adhesion. Exp Cell Res 292: 295–303. 14697337 10.1016/j.yexcr.2003.08.021

[5] Vearing C, Lee FT, Wimmer-Kleikamp S, Spirkoska V, To C, Stylianou C, et al (2005) Concurrent binding of anti-EphA3 antibody and ephrin-A5 amplifies EphA3 signaling and downstream responses: potential as EphA3-specific tumor-targeting reagents. Cancer Res 65:6745–6754. 16061656 10.1158/0008-5472.CAN-05-0758

[6] Pasquale EB (2010) Eph receptors and ephrins in cancer: bidirectional signalling and beyond. Nat Rev Cancer 10: 165–180. 10.1038/nrc2806 20179713 PMC2921274

[7] Wu R, Wang H, Wang J, Wang P, Huang F, Xie B, et al (2014) EphA3, induced by PC-1/PrLZ, contributes to the malignant progression of prostate cancer. Oncol Rep 32:2657–2665. 10.3892/or.2014.3482 25231727

[8] Zhao YL, Wu RQ, Li HL (2012) Inhibition effect of 1504-siRNA on EhpA3 expressed HCT116 cells. Mil Med Sci 36: 272–275.

[9] Zhao YL, Wu RQ, Li HL (2012) Inhibition effect of 1504-siRNA on EhpA3 expressed HGC27 cells. Lett in Biotech 23: 503–505.

[10] Zou W, Luo C, Zhang Z, Liu J, Gu J, Pei Z, et al (2004) A novel oncolytic adenovirus targeting to telomerase activity in tumor cells with potent. Oncogene 23: 457–464. 14724574 10.1038/sj.onc.1207033

[11] Yu ST, Chen L, Wang HJ, Tang XD, Fang DC, Yang SM (2009) hTERT promote the invasion of telomerase-negative tumor cells in vitro. Int J Oncol 35: 329–336. 19578747

[12] Liu HY, Han BJ, Zhong YX, Lu ZZ (2009) A three-plasmid system for construction of armed oncolytic adenovirus. J Virol Methods. 162: 8–13. 10.1016/j.jviromet.2009.07.011 19646479

[13] Nyberg-Hoffman C, Shabram P, Li W, Giroux D, Aguilar-Cordova E (1997) Sensitivity and reproducibility in adenoviral infectious titer determination. Nat. Med 3: 808–811. 9212113 10.1038/nm0797-808

[14] Zhuang G, Song W, Amato K, Hwang Y, Lee K, Boothby M, et al (2012) Effects of cancer-associated EphA3 mutations on lung cancer. J Natl Cancer Inst 104:1182–1197. 10.1093/jnci/djs297 22829656 PMC3611812

[15] Hu ZB, Wu CT, Wang H, Zhang QW, Wang L, Wang RL, et al (2008) A simplified system for generating oncolytic adenovirus vector carrying one or two transgenes. Cancer Gene Ther 15: 173–182. 18157145 10.1038/sj.cgt.7701105

[16] Meerani S, Yao Y (2010) Oncolytic Viruses in Cancer Therapy.Euro J Sci Res 40: 156–171.

[17] Logue SE, Martin SJ. (2008) Caspase activation cascades in Apoptosis. Biochem Soc Trans 36:1–9. 10.1042/BST0360001 18208375

[18] Ito H, Aoki H, Kühnel F, Kondo Y, Kubicka S, Wirth T, et al (2006) Autophagic cell death of malignant glioma cells induced by a conditionally replicating adenovirus. J Natl Cancer Inst 98: 625–636. 16670388 10.1093/jnci/djj161

[19] Yokoyama T, Iwado E, Kondo Y, Aoki H, Hayashi Y, Georgescu MM, et al (2008) Autophagy-inducing agents augment the antitumor effect of telerase-selve oncolytic adenovirus OBP-405 on glioblastoma cells. Gene Ther 15: 1233–1239. 10.1038/gt.2008.98 18580968

[20] Qu X, Yu J, Bhagat G, Furuya N, Hibshoosh H, Troxel A, et al (2003) Promotion of tumorigenesis by heterozygous disruption of the beclin1 autophagy gene. J Clin Invest 112: 1809–1820. 14638851 10.1172/JCI20039 PMC297002

[21] Liang C, Feng P, Ku B, Dotan I, Canaani D, Oh BH, et al (2006) Autophagic and tumour suppressor activity of a novel Beclin1-binding protein UVRAG. Nat Cell Biol 8: 688–699. 16799551 10.1038/ncb1426

[22] Nihira K, Miki Y, Ono K, Suzuki T, Sasano H (2014) An inhibition of p62/SQSTM1 caused autophagic cell death of several human carcinoma cells. Cancer Sci 105:568–575. 10.1111/cas.12396 24618016 PMC4317843

