# Supplementary figures and images for: Antitumor Effects of Oncolytic Adenovirus-Carrying siRNA Targeting Potential Oncogene EphA3
Source: PLoS One. 2015 May 15;10(5):e0126726. doi: 10.1371/journal.pone.0126726 (PMC4433270; doi:10.1371/journal.pone.0126726)

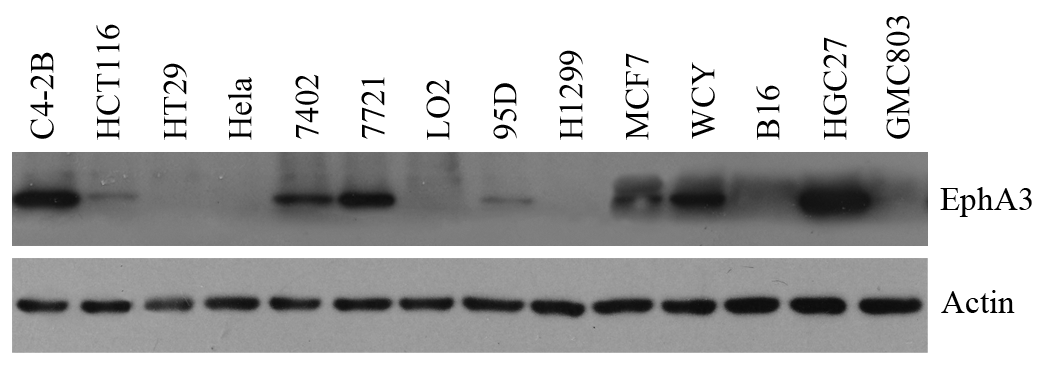

Supplement: S1 Fig — To select EphA3-highly expressing cell lines, EphA3 protein was measured among fourteen cell lines of seven tumor types, C4-2B, LNCaP of prostate cancer, HGC27, MGC803 of gastric carcinoma, WCY, MCF7 of mammary adenocarcinoma, H1299, 95D of lung cancer, SMMC-7721, Bel-7402 of hepatoma carcinoma, Hela of cervical cancer, HT29, HCT116 of colon carcinoma originating from human, B16 of melanoma originating from mice and one normal cell line, LO2. After three batches of experiments C4-2B, HCT116, Bel-7402, SMMC-7721, MCF7, WCY, HGC27 cell lines were confirmed to have more EphA3 protein (S1 Fig). No lung cancer cell lines highly expressed EphA3, confirming that EphA3 was a tumor suppressor gene in lung cancer [17]. (TIF) [file pone.0126726.s001.tif]

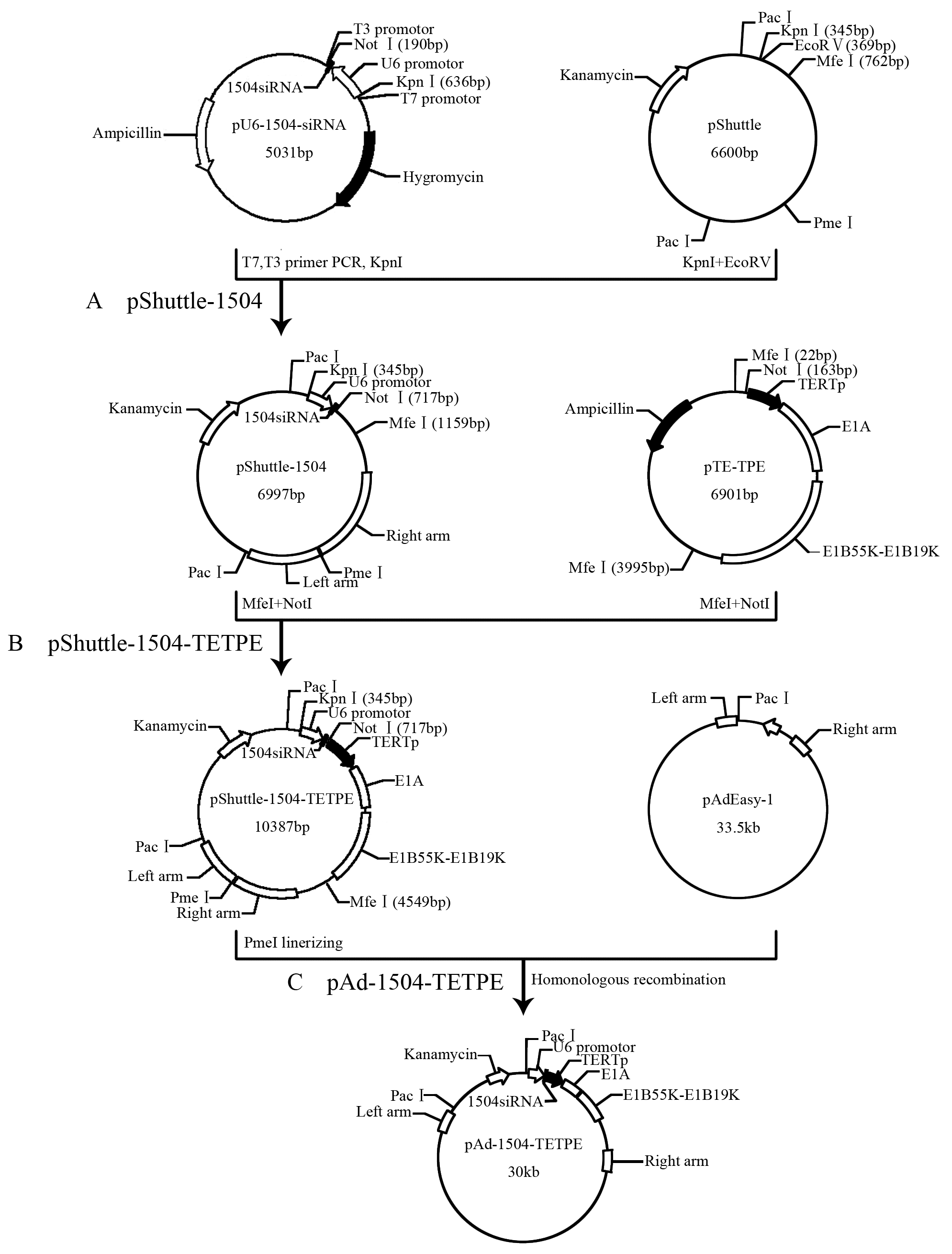

Supplement: S2 Fig — (A) Plasmid maps of pshuttle-1504, pU6-1504-siRNA, and pshuttle. 1504-siRNA was amplified by PCR with pU6-1504-siRNA as the template using T7 and T3 primers. PCR production cut with KpnI and pshuttle cut with KpnI and EcoRI were ligated to produce pshuttle-1504 that contains 1504-siRNA (5’GCG GTC AGC ATC ACA ACT AAT 3’). (B) Plasmid maps of pshuttle-1504-TETPE and pTE-TPE. pTE-TPE containing the total E1 region except for the promoter of E1A and the promoter of telomerase reverse transcriptase (TERTp) and pshuttle-1504 were all digested with MfeI and NotI and then recovered portions were ligated to generate pshuttle-1504-TETPE which contains 1504-siRNA and the TERTp driven E1A region. (C) Plasmid Maps of pAdEasy-1 and pAd-1504-TETPE. pshuttle-1504-TETPE linearized with PmeI and pAdEasy were mixed and cotransformed into competent BJ5183 cells to produce pAd-1504-TETPE, which contains 1504-siRNA, TERTp, E1A and an Ad backbone. (TIF) [file pone.0126726.s002.tif]
